# Insulin mimetics and non-canonical insulin receptor signaling: mechanisms and disease relevance

**DOI:** 10.1038/s12276-026-01862-5

**Published:** 2026-10-07

**Authors:** Junhee Park, Eunhee Choi

**Affiliations:** 1 Department of Oral Histology and Developmental Biology, School of Dentistry, Seoul National University, Seoul, Republic of Korea; 2 Department of Pathology and Cell Biology, Vagelos College of Physicians and Surgeons, Columbia University, New York, NY USA

**Keywords:** Metabolism, Mechanisms of disease, Drug discovery, Insulin signalling

## Abstract

Insulin receptor (IR) signaling has traditionally been viewed as a canonical pathway in which insulin binding uniformly activates downstream metabolic and mitogenic responses. However, growing evidence indicates that IR signaling is not monolithic. Instead, IR signaling is increasingly recognized as a context-dependent process in which receptor conformation, spatial organization, and intracellular trafficking shape signaling outcomes. These non-canonical regulatory mechanisms generate distinct signaling states that can differentially regulate metabolic, mitogenic, immune, and neuronal functions. Recent advances in engineered insulin mimetics, including peptides, antibodies, aptamers, and de novo designed ligands, have provided powerful tools to probe this signaling diversity. By stabilizing specific receptor conformations or engagement geometries, these ligands can selectively bias downstream signaling programs. Here we discuss recent insights into how the qualitative features of IR signaling, rather than signaling amplitude alone, determine disease outcomes across diverse pathological contexts. Understanding this signaling diversity may enable the rational design of next-generation insulin mimetics and more precise therapeutic strategies targeting IR pathways.

## Insulin receptor signaling reveals context-dependent mechanisms

The discovery of insulin in the 1920s revolutionized diabetes treatment, transforming it from a fatal disease to a manageable condition. This review explores the insulin receptor (IR) signaling, which extends beyond glucose regulation to influence neuronal activity, immune responses, and kidney function. The authors discuss how IR signaling is not solely dependent on receptor activation but also on qualitative features like pathway selection and receptor localization. They highlight that insulin and its mimetics can stabilize distinct receptor states, leading to varied signaling outcomes. The study reveals that engineered ligands can modulate IR signaling, offering potential therapeutic avenues for diseases such as diabetes, cancer, and Alzheimer disease (AD). These findings suggest that future research could focus on developing insulin mimetics with improved therapeutic profiles, potentially leading to more precise treatments for insulin-related disorders.

## Introduction

The discovery of insulin in the early 1920s marked a turning point in modern medicine, transforming diabetes from a fatal disease into a manageable condition^1,2^. The subsequent identification of the insulin receptor (IR) provided the molecular basis for insulin action at the cellular level^3–5^. Early biochemical studies demonstrated the presence of specific, high-affinity insulin-binding sites on the cell surface, leading to the recognition of the IR as a receptor tyrosine kinase (RTK) capable of ligand-induced autophosphorylation and downstream signaling. These discoveries fueled decades of work mapping canonical IR-dependent signaling pathways, the PI3K–AKT and MAPK axes^6^, which became the prevailing framework for understanding insulin action.

As IR signaling pathways were progressively elucidated, it became clear that insulin exerts biological effects extending well beyond classical metabolic regulation. In the brain, IR signaling controls neuronal activity, synaptic plasticity, and cognition^7^. In immune cells, IR signaling modulates immune activation, inflammatory responses, and immune metabolism^8^, and in the kidney, it contributes to epithelial function and tissue homeostasis^9^. Together, these findings indicate that IR signaling operates across diverse tissues and cell types, supporting tissue-specific physiological functions beyond glucose control.

Consistent with this expanded biological role, dysregulation of IR signaling has been implicated in a broad spectrum of pathological conditions. In addition to diabetes and severe insulin resistance syndromes, altered IR signaling contributes to the pathogenesis of cancer^10^, chronic inflammation and immune dysregulation^11^, and kidney diseases^12^. Notably, hyperinsulinemia and context-dependent signaling biases potentially shape disease progression, challenging classical models of insulin action.

These observations motivate a conceptual shift in how IR signaling is understood. Emerging evidence suggests that IR signaling outcomes are determined not only by the extent of receptor activation but also by qualitative features of the signal, including pathway selection, temporal dynamics, subcellular localization, and ligand-specific receptor engagement. Analogous to biased agonism described for G-protein-coupled receptors, insulin and alternative IR ligands can stabilize distinct receptor states that lead to non-canonical signaling outputs.

**Fig. 1:**
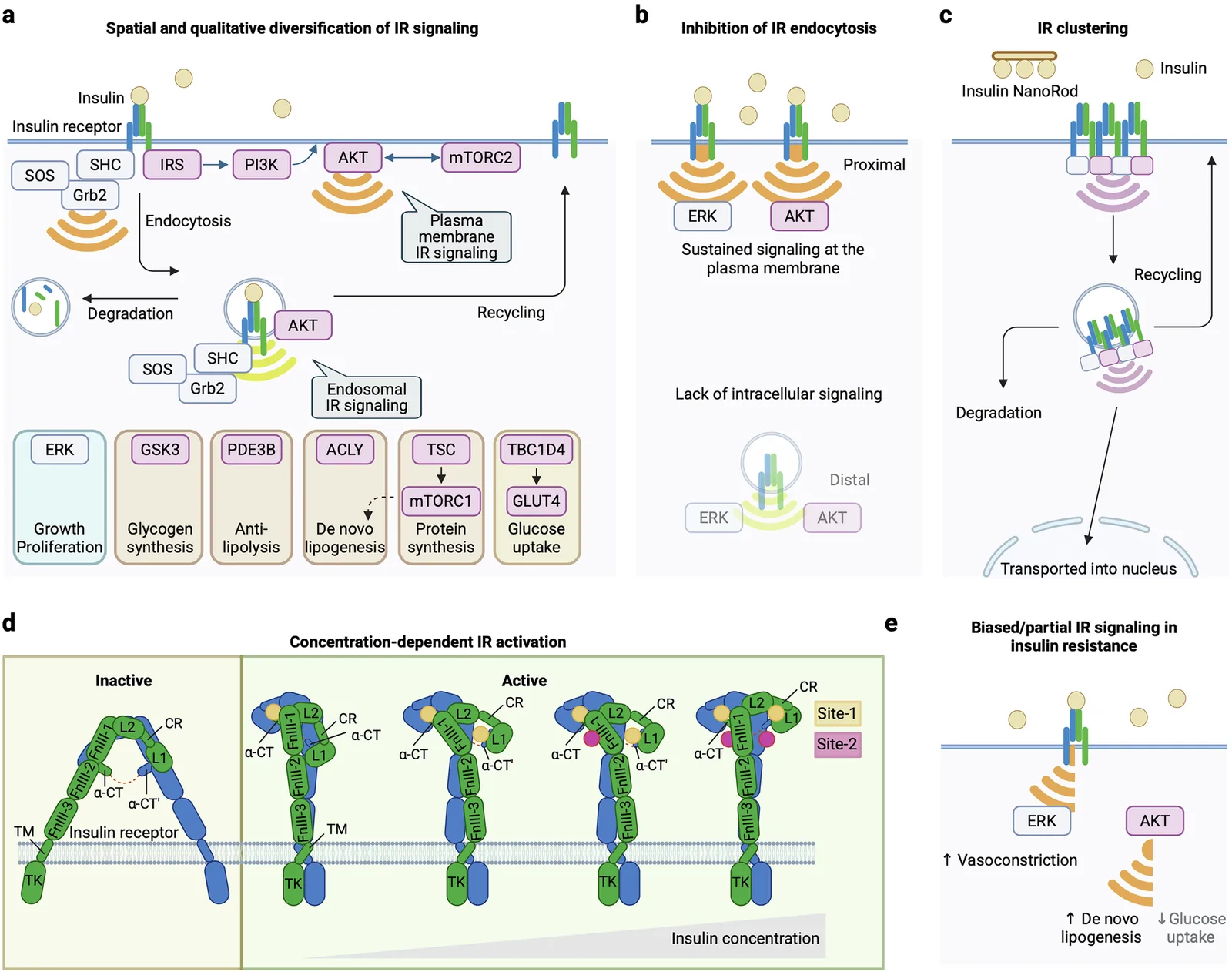
Regulation of insulin receptor signaling. **a** At the plasma membrane, insulin binds to and activates IR, triggering two major signaling pathways: the PI3K–AKT pathway and the MAPK–ERK pathway, which regulate metabolism as well as cell growth and proliferation. Activated IR undergoes endocytosis, contributing to the spatial and temporal regulation of IR signaling. PI3K–AKT-associated metabolic outcomes are highlighted in orange, whereas MAPK–ERK-associated growth and mitogenic outcomes are highlighted in blue. **b** Inhibition of IR endocytosis prolongs plasma membrane IR signaling while reducing intracellular IR signaling. IR endocytosis is a major pathway for insulin clearance; therefore, inhibition of IR endocytosis leads to hyperinsulinemia. Systemic inhibition of IR endocytosis also alters lipid metabolism. **c** IR forms clusters at the plasma membrane and within intracellular compartments, including the cytosol and nucleus, thereby contributing to the spatial and temporal regulation of IR signaling. **d** IR can adopt multiple active conformations depending on insulin occupancy, which may fine-tune IR signaling under different insulin concentrations. IR protomers are shown in green and blue. Red dotted lines indicate disulfide bonds between α-chain C-terminal (α-CT) segments. Yellow denotes site-1 insulin, and purple denotes site-2 insulin. **e** Under insulin-resistant conditions, IR signaling pathways exhibit differential sensitivity to insulin, resulting in pathway-selective or partial IR signaling. In endothelial cells, impairment of the PI3K–AKT–eNOS pathway reduces nitric oxide-dependent vasodilation, whereas relatively preserved MAPK signaling can promote vasoconstriction. In metabolic tissues, selective insulin resistance can also uncouple distinct insulin-regulated processes. For example, impaired insulin-stimulated GLUT4 translocation reduces glucose uptake in skeletal muscle and adipose tissue, whereas hepatic lipogenic signaling can remain relatively preserved, contributing to de novo lipogenesis despite impaired suppression of hepatic glucose production. Abbreviations: ACLY ATP citrate lyase, eNOS endothelial nitric oxide synthase, FnIII-1 first fibronectin type III domain, FnIII-2 second fibronectin type III domain, FnIII-3 third fibronectin type III domain, GLUT4 glucose transporter type 4, L1 leucine-rich repeat domain 1, mTORC1 mechanistic target of rapamycin complex 1, mTORC2 mechanistic target of rapamycin complex 2, PDE3B phosphodiesterase 3B, SHC Src homology 2 domaincontaining transforming protein, TBC1D4 TBC1 domain family member 4, TK tyrosine kinase domain, TM transmembrane domain, TSC tuberous sclerosis complex.

In this review, we focus on how diverse modes of IR activation generate distinct signaling programs with important implications for disease biology. We discuss emerging concepts of non-canonical and biased IR signaling, including spatially and temporally compartmentalized signaling from plasma membrane-proximal, endosomal, and higher-order clustered receptor states (Fig. 1a–c). Building on this framework, we examine how insulin mimetics serve not only as therapeutic candidates but also as powerful tools to dissect and selectively modulate IR signaling in physiological and pathological contexts. To clarify the terminology used throughout this review, we have provided working definitions of key concepts in non-canonical IR signaling in Box 1.

Box 1 Working definitions of key terms in non-canonical insulin receptor signaling

**Table:** 

Term	Definition
Non-canonical IR signaling	Signaling outputs that arise from qualitative features of IR regulation beyond classical ligand-induced activation, including altered receptor conformation, signaling bias, spatial organization, and trafficking.
Partial agonism	Reduced maximal receptor activation or downstream signaling relative to insulin.
Biased agonism	Preferential activation of one signaling pathway over another compared with insulin.
Spatially compartmentalized signaling	Signaling from distinct receptor pools, such as the plasma membrane, endosomal clusters, or other subcellular IR populations.
Trafficking-dependent signaling	Signaling outcomes influenced by receptor endocytosis, recycling, degradation, or compartment residence time.

## Regulatory mechanism of insulin receptor signaling

Growing evidence indicates that IR signaling outcomes are determined not only by the magnitude of receptor activation but also by how signaling is organized across multiple regulatory dimensions. In particular, pathway selection, temporal dynamics of receptor activation, and spatial organization of receptor complexes can shape downstream signaling outputs. Processes such as receptor clustering and internalization into endosomal compartments influence signal duration, pathway engagement, and cellular responses^13,14^. These regulatory layers extend beyond ligand binding and receptor phosphorylation and help explain how qualitative features of IR signaling, rather than signaling magnitude alone, can generate distinct biological outcomes.

### Canonical insulin-dependent activation of the insulin receptor

IR is an RTK whose activation is governed by ligand-induced conformational rearrangements. Unlike many RTKs that undergo ligand-induced dimerization, IR exists as a constitutive dimer at the cell surface^15^ (Fig. 1d). In the ligand-free state, the receptor adopts an autoinhibited conformation in which the ectodomain geometry enforces separation and unfavorable orientation of the intracellular tyrosine kinase domains, thereby suppressing spontaneous trans-phosphorylation^16,17^.

Insulin-induced activation follows a multistep binding trajectory characterized by intrinsic allostery and ligand occupancy-dependent conformational progression^18,19^ (Fig. 1d). Insulin engages the receptor through two binding sites, site-1 and site-2. Site-1 is the primary insulin- binding site formed by the leucine-rich repeat domain 1 (L1) of one protomer together with the α-chain C-terminal (α-CT) segment and the loop of the first fibronectin type III domain (FnIII-1) from the opposing protomer^20^. By contrast, site-2 is located on the lateral surface of FnIII-1.

In the fully liganded active state, the receptor can accommodate up to four insulin molecules (Fig. 1d). This multivalent engagement promotes and stabilizes the T-shaped conformation of the receptor through coordinated ligand–receptor and receptor–receptor interactions, thereby promoting juxtaposition of the intracellular tyrosine kinase domains and facilitating efficient trans-autophosphorylation^19^. The detailed mechanisms underlying insulin-dependent IR activation have been extensively reviewed^21,22^. Here we provide an overview to establish a framework for comparison with non-canonical models of receptor activation discussed below.

Once the tyrosine kinase domains are juxtaposed, trans-autophosphorylation proceeds on multiple tyrosine residues within the activation loop, juxtamembrane region, and C-terminal tail^23^. Activation loop phosphorylation stabilizes the active catalytic conformation and increases kinase activity^24^, whereas additional phosphorylation events generate a structured docking platform for downstream adaptor proteins^25,26^. In particular, the phosphorylation of specific juxtamembrane and C-terminal tyrosine residues promotes recruitment of insulin receptor substrate (IRS) proteins, which function as primary scaffolds linking the activated receptor to PI3K signaling^27,28^ (Fig. 1a). IRS protein engagement enables the association of the p85 regulatory subunit of PI3K, leading to activation of the PI3K–AKT pathway and downstream regulation of glucose transport, glycogen synthesis, lipid metabolism, and protein synthesis^28,29^.

In parallel with IRS-dependent signaling, the activated IR can recruit the adaptor protein Src homology 2 domain-containing transforming protein (Shc) through phosphorylation of alternative tyrosine residues^30^ (Fig. 1a). Shc binding facilitates the assembly of the Grb2–SOS complex and subsequent activation of the MAPK cascade, which is more closely associated with mitogenic and proliferative outputs^31,32^. Importantly, IRS- and SHC-mediated signaling pathways are not simply parallel branches but represent competing adaptor engagement routes; their relative utilization shapes the overall downstream IR signaling balance^33^.

### Non-canonical insulin receptor signaling

#### Endosomal signaling and receptor trafficking

Activated IR is internalized into endocytic compartments, where insulin dissociates from the receptor and is targeted for lysosomal degradation (Fig. 1a). The insulin-free receptor subsequently recycles back to the plasma membrane for another round of signaling or is directed toward lysosomal degradation, thereby limiting receptor availability at the cell surface. Within this canonical framework, endocytosis functions primarily to constrain the duration and magnitude of IR signaling by terminating ligand engagement and regulating cellular sensitivity to insulin.

Accumulating evidence indicates that internalized IR can remain signaling-competent within endosomal compartments, where adaptors and phosphorylated receptors may continue to influence downstream pathways^34,35^. Recent proteomic analysis demonstrates that plasma membrane-proximal IR signaling remains intact, whereas distal IR signaling is defective in the skeletal muscle of patients with type 2 diabetes, suggesting that spatial distribution of receptor signaling contributes to disease pathogenesis^36^. In addition, insulin-stimulated AKT2 phosphorylation has been shown to depends on WD repeat and FYVE domain-containing protein 2 (WDFY2)-enriched endosomal compartments, indicating that endosomal localization can confer qualitative specificity on IR signaling outputs rather than simply diminish signal strength^37^.

Genetic models further demonstrate that IR trafficking has direct physiological consequences for systemic metabolic regulation (Fig. 1b). Mitotic arrest deficient 2 (MAD2), a spindle checkpoint protein, also regulates insulin-stimulated IR endocytosis through direct interaction with IR^38^. In mice, disruption of the IR–MAD2 interaction delays insulin-activated IR endocytosis, reduces insulin clearance, and elevates circulating insulin levels. This prolongs plasma membrane-associated IR signaling and may alter signaling from internalized receptor pools, thereby reshaping the spatial and temporal properties of IR signaling. Consistent with this model, impaired IR endocytosis alters metabolic homeostasis, including changes in lipid metabolism^38^. Conversely, the genetic ablation of p31^comet^, an antagonist of MAD2, leads to reduced basal plasma membrane IR abundance and increased intracellular IR localization, producing metabolic phenotypes similar to those observed in IR-deficient mice^39^. Comparable in vivo observations have been reported for additional trafficking regulators in genetic models. Hepatic overexpression of Eph receptor B4 (EphB4), an RTK involved in cell–cell communication, promotes IR endocytosis and lysosomal degradation, reduces receptor abundance, and induces insulin resistance, whereas hepatic EphB4 deficiency improves systemic insulin sensitivity^40^. In parallel, β-cell-specific loss of the IR scavenger inceptor increases IR activation and improves glucose tolerance^41^. These in vivo studies demonstrate that precise control of IR endocytosis is required for systemic metabolic homeostasis.

#### Receptor clustering and spatial organization

IR signaling is also shaped by receptor organization at the plasma membrane, which represents a mechanistically distinct layer of spatial regulation. IR clustering and nanoscale organization can modulate receptor activation and downstream signaling outputs^42–45^(Fig. 1c). Disease-associated cellular conditions, including oxygen stress, can perturb receptor clustering dynamics^13^, providing an additional mechanism by which the spatial organization of IR signaling may be altered independently of receptor internalization.

#### Pathway bias and temporal regulation

IR signaling outputs are further shaped by pathway selection and temporal dynamics (Fig. 1e). IR activation engages two major signaling pathways, PI3K–AKT and MAPK, which can become selectively distorted in insulin-resistant states. For example, in the vasculature, AKT signaling is impaired, whereas MAPK signaling is relatively preserved; this leads to reduced nitric oxide production and enhanced ERK activation, thereby contributing to endothelial dysfunction^46^. Temporal patterns of insulin exposure are differentially interpreted by downstream signaling networks. Transient versus sustained activation of the PI3K–AKT axis leads to differential regulation of metabolic targets. In hepatocyte models, S6K preferentially responds to transient AKT activation, G6Pase integrates sustained AKT signaling, and GSK3β responds to both, illustrating how temporal features of IR signaling can differentially route metabolic outputs^47^. Notably, pulsatile insulin delivery elicits stronger hepatic IR signaling than constant insulin exposure despite equivalent insulin dose, highlighting the importance of temporal insulin dynamics in determining signaling efficacy^48^.

Together, these observations highlight that IR signaling outcomes are determined not only by receptor activation but also by spatial organization, pathway selection, and temporal dynamics, providing a framework for understanding how qualitative features of IR signaling shape biological responses.

### Concentration-dependent insulin receptor activation

Structural and functional analyses reveal that IR can adopt multiple receptor conformations depending on ligand occupancy, giving rise to structurally distinct activation states (Fig. 1d)^19,49^. Incomplete ligand occupancy produces alternative conformations: binding of a single insulin molecule stabilizes gamma-shaped structure, whereas two to three bound insulin molecules promote an asymmetric T-shaped configuration. By contrast, full ligand saturation favors a symmetric T-shaped conformation, suggesting that ligand-dependent conformational heterogeneity is an intrinsic feature of IR activation rather than a transition to a single uniform signaling state^50^.

Despite these structural differences, the membrane-proximal regions of the insulin-bound receptor remain positioned sufficiently close to enable efficient trans-autophosphorylation of the intracellular kinase domains^50^. Site-specific insulin mutant studies further suggest that the symmetric T-shaped conformation may represent a more optimally active state compared with the asymmetric configuration. However, whether these structurally distinct extracellular conformations differentially influence kinase domain arrangement, stability, autophosphorylation kinetics, downstream pathway engagement, or receptor trafficking remains unclear. Moreover, which conformational states predominate in vivo, particularly under distinct physiological or pathophysiological conditions, remains to be elucidated.

## Insulin mimetics as signaling modulators

Engineered ligands have provided powerful tools to interrogate IR structure–function relationships and signaling mechanisms. These molecules can disrupt autoinhibitory conformations, stabilize distinct active states, or promote higher-order receptor organization, thereby altering signaling output. Here we examine insulin mimetics not simply as insulin substitutes, but as molecular probes that reveal non-canonical modes of IR activation. By engaging the receptor through alternative binding geometries or valencies, these ligands expose signaling states not readily accessible through native insulin alone. We do not discuss engineered insulin variants or naturally occurring insulin-like peptides from other species; instead, we focus on artificial ligands specifically designed to modulate IR activation, signaling bias, and trafficking dynamics. To improve readability and to distinguish mechanistic promise from translational feasibility, Table 1 summarizes the IR mimetics and modulators discussed in this section.

**Table 1:** Insulin receptor regulators: mechanism of receptor engagement, signaling outputs, and developability considerations.

Class / representative examples	Mode of receptor engagement	Pharmacological action / signaling output	Reported biological effect	Potential translational advantages	Developability liabilities and practical implication
**Synthetic peptide** **S519, S597**	Bivalent site-2–site-1 fusion peptides. S597 engages L1 and FnIII-1 domains of IR and stabilizes an extended T-shaped IR conformation.	Partial or biased signaling relative to insulin; preferential AKT over ERK signaling.	S597 lowers glucose levels in vivo, activates insulin-binding deficient disease-causing IR mutants, and shows selectivity for IR over IGF1R.	Relatively modular; chemically synthesizable; smaller than antibodies or DNA scaffolds; useful as structural and signaling probes.	Likely require parenteral administration; may have short half-life due to proteolysis and renal clearance; half-life extension could alter tissue distribution, receptor residence, trafficking, or signaling bias. Most plausible near-term use is as injectable peripheral agents after stability and exposure optimization. Potential immunogenicity of non-natural sequences.
**Synthetic peptide** **S661, S961**	Bivalent site-1–site-2 fusion peptides that stabilize inactive or intermediate IR conformation.	Antagonist	Inhibits insulin-dependent IR activation and downstream signaling; useful tools for probing insulin action and hyperinsulinemic states.		
**Computationally designed proteins** **S2-F1-S1, RF-405, RF-409**	De novo designed site-2 and site-1 binders fused with tunable linker geometry and flexibility; designed proteins can impose non-native receptor geometries and stabilize extended T-shaped IR conformation.	Tunable agonists ranging from biased or trafficking-altered signaling (S2-F1-S1) to more balanced insulin-like activation (RF-405, RF-409), depending on linker geometry and rigidity.	Reported to induce glucose lowering in vivo, retain activity on disease-causing IR mutants, and produce distinct ERK, trafficking, and proliferation outputs depending on design. Selectivity for IR over IGF1R.	High structural programmability; can encode non-native binding geometries, valency, and spacing; strong mechanistic value for dissecting IR conformational signaling.	Likely parenteral; potential immunogenicity of non-natural sequences; aggregation, solubility, stability, formulation, analytical characterization, and scalable manufacturing under good manufacturing practice (GMP) standards must be assessed early. Therapeutic translation requires developability screening; immunogenicity assessment, and pharmacokinetic/pharmacodynamic engineering.
**Computationally designed proteins** **S1–S2-type designs**	Designed site-1 and site-2 binders arranged to trap IR in inactive or intermediate conformations.	Antagonists	Inhibits insulin-dependent IR activation and downstream signaling.		
**DNA aptamer** **IR-A43**	Binds an allosteric site and stabilizes an insulin-bound asymmetric receptor conformation.	Insulin-dependent positive allosteric modulator.	Potentiates insulin-stimulated IR activation without independently activating the receptor.	Chemically programmable; can be multivalent; potentially reversible using complementary oligonucleotides that neutralize aptamer activity.	Susceptible to nuclease degradation and rapid renal clearance unless chemically modified or conjugated; tissue distribution, penetration, and repeated-dosing safety require careful evaluation. Systemic use would require stabilization, half-life extension, and biodistribution optimization.
**DNA aptamer** **IR-A62**	In the absence of insulin, engages site-1 and site-2 and induces an arrowhead-shaped IR.	Context-dependent allosteric modulator with partial agonist activity.	Preferentially induces Y1150 monophosphorylation and metabolic signaling; enhances or inhibits insulin-dependent signaling depending on concentration		
**Anti-IR antibodies** **83-7, 83-14**	Bind extracellular IR epitopes, including CR or FnIII-1 domains, and can activate receptors through antibody-mediated receptor engagement or cross-linking.	Partial agonist-like activity with preferential AKT activation.	Improve glucose tolerance or hyperinsulinemia in receptoropathy models, but receptor downregulation may attenuate the beneficial effect.	Long systemic half-life; high specificity; established antibody manufacturing and regulatory path; can impose receptor clustering or cross-linking geometries.	Injectable; limited tissue penetration; possible receptor downregulation. Attractive for peripheral severe insulin resistance syndromes, but the therapeutic window may be limited by competing receptor activation and receptor loss. Long half-life can be an advantage but also creates safety concerns if excessive or prolonged IR activation occurs. Most relevant for peripheral metabolic indications rather than central nervous system (CNS) indications unless blood–brain barrier (BBB) delivery is engineered.
**Anti-IR antibodies** **XMetA**	Allosteric antibody-mediated activation of IR.	Partial agonist with preferential AKT activation.	Lower glucose levels and improves metabolic parameters in diabetic animal models without strong mitogenic responses.		
**Anti-IR antibodies** **XMetS**	Positive allosteric modulation of insulin binding and insulin-stimulated signaling.	Insulin-sensitizing positive allosteric modulator.	Increases insulin binding affinity and potentiates insulin-stimulated IR signaling; improves glucose metabolism in vitro and in vivo.		
**Anti-IR antibodies** **XMetD**	Negative allosteric modulation of insulin binding and signaling	Allosteric antagonist or negative allosteric modulator	Attenuates insulin-dependent IR activation and signaling and reverses hypoglycemia in hyperinsulinemic mouse models.		
**Insulin–DNA origami NanoRods**	Multivalent insulin presentation with defined ligand spacing and valency.	Valency- and spacing-dependent enhancement of IR binding, residence time, and activation.	Increases receptor residence time and enhances IR autophosphorylation and downstream signaling responses; multivalent constructs reduced glucose in Zebrafish model.	Exceptional control over ligand spacing, valency, and receptor-clustering geometry; powerful platform for testing spatial regulation of IR signaling.	Mostly preclinical; challenges include serum stability, innate immune activation, biodistribution, tissue penetration, BBB access for CNS indications, scalable GMP manufacturing, and route of administration.

### Insulin mimetic peptides

Early phage display screens identified two spatially distinct ligand-binding “hotspots” on IR, termed site-1 and site-2^51–54^. Synthetic peptides targeting these regions were subsequently fused in different orientations, generating ligands that function as partial agonists or antagonists depending on topology.

#### Agonists (site-2–site-1 fusion)

**Fig. 2:**
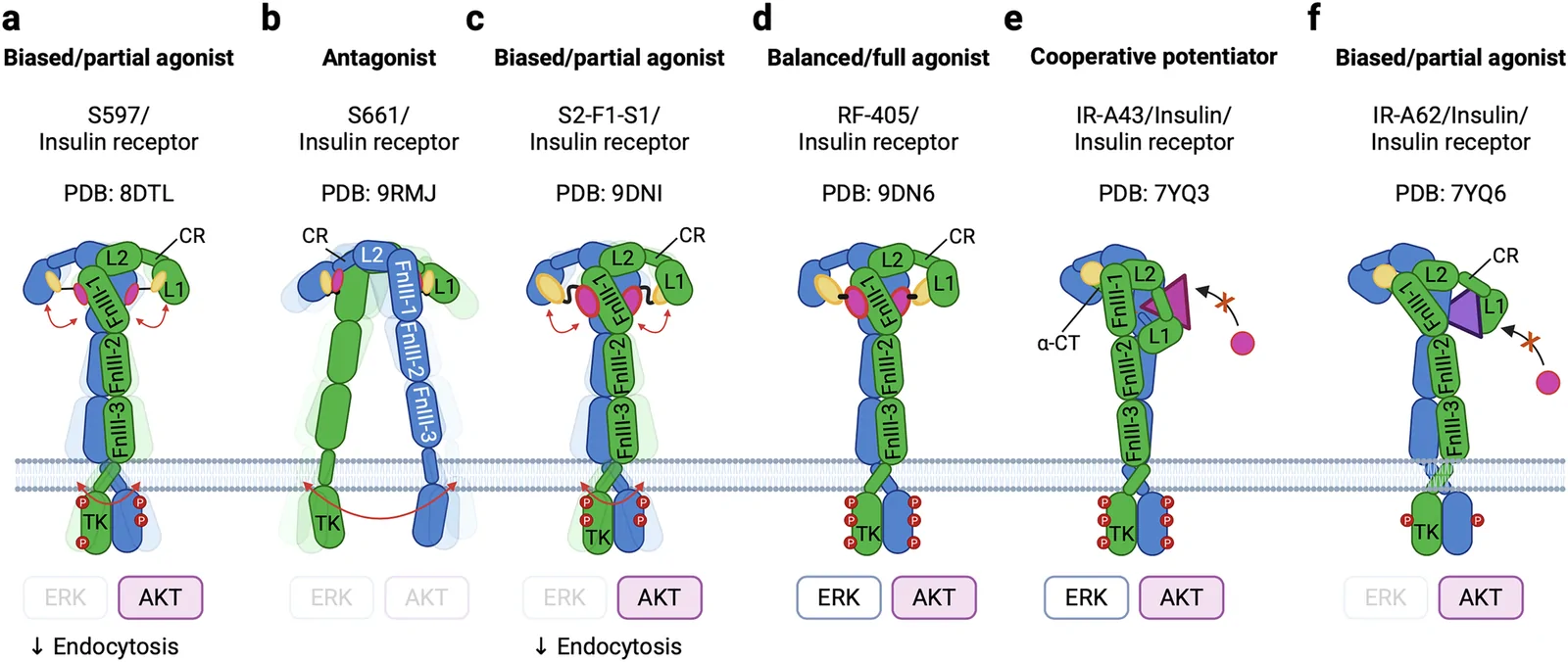
Non-canonical insulin receptor activation by engineered ligands. Engineered ligands bind to IR, induce conformational changes, and stabilize distinct active conformations of the receptor. IR protomers are shown in green and blue. Yellow denotes the site-1-binding component or site-1 insulin, and purple denotes the site-2-binding component or site-2 insulin. The black line between site-1 and site-2 components indicates the linker connecting the two components. Triangles represent aptamers (IR-A43 and IR-A62). **a** S597 disrupts the autoinhibitory conformation of IR and induces a conformational transition to an extended T-shaped structure, enabling trans-autophosphorylation. The flexible linker of S597 promotes a more dynamic receptor conformation that is less efficient at stabilizing the kinase alignment, resulting in partial or biased activation of downstream signaling. **b** S661 binds the site-1 and site-2 surfaces of IR and disrupts the autoinhibitory conformation. However, S661 fails to stabilize the active conformation and therefore does not support productive signaling, functioning as an antagonist. **c**, **d** S2-F1-S1 (**c**) and RF-405 (**d**) share the same site-1 and site-2 components but differ in linker flexibility. S2-F1-S1 contains a flexible linker, whereas RF-405 contains a rigid linker. Both ligands engage two IR protomers simultaneously and induce an extended T-shaped conformation. However, the flexible linker in S2-F1-S1 results in a more dynamic active state, leading to partial or biased signaling toward the AKT pathway, whereas RF-405 stabilizes a compact and fully active conformation that supports balanced signaling, similar to insulin. **e**, **f** IR-A43 (**e**) and IR-A62 (**f**) do not disrupt insulin binding at site-1 but interfere with insulin binding at site-2. In the presence of insulin, IR-A43 stabilizes a gamma-shaped asymmetric active conformation, supporting both AKT and ERK signaling. Conversely, IR-A62 stabilizes a less stable receptor intermediate by mimicking site-1/site-2′ insulin coordination, resulting in preferential signaling toward the AKT pathway.

S519, a 36-amino acid site-2–site-1 fusion peptide, demonstrated that multivalent engagement could activate IR through non-native geometries^54^. Optimization yielded S597, a 31-amino acid derivative with improved affinity. S597 robustly promotes receptor autophosphorylation while preferentially activating metabolic pathways and relatively attenuating mitogenic signaling, thereby indicating partial or biased agonism (Fig. 2a). Functional studies further revealed that S597 elicited metabolic responses, and in vivo analyses suggested potential physiological benefits of biased IR signaling in atherosclerosis^52,55–57^.

Structural studies performed nearly two decades after the initial hotspot discovery clarified the molecular basis of these effects^57,58^. The site-1 component of S597 functionally replaces the α-CT and directly engages the L1 domain, whereas the site-2 component binds the FnIII-1 domain, analogous to insulin at site-2^57^ (Fig. 2a). Two molecules of S597 bind per IR dimer, with each peptide bridging site-1 and site-2 across two protomers, thereby cross-linking and stabilizing a distinct receptor configuration. Despite this non-native binding geometry, the membrane-proximal regions of the S597-bound IR are positioned sufficiently close to enable efficient trans-autophosphorylation, indicating that this conformation represents an active signaling state. These findings demonstrated that alternative binding topologies can stabilize non-canonical active conformations.

#### Antagonists (site-1–site-2 fusion)

Reversing the fusion orientation yielded antagonists such as S661 and its optimized derivative S961^59^. These peptides inhibit insulin-stimulated IR activation and downstream metabolic signaling despite sharing nearly identical binding elements with agonists.

Recent cryo-electron microscopy (cryo-EM) structures of IR bound to S661 and related derivatives provide important insights into this paradox^60^ (Fig. 2b). In these complexes, the receptor adopts an intermediate conformation positioned between the inactive and active states. The ligand engages the receptor in a manner similar to S597, but its fusion orientation and linker geometry restrict the conformational rearrangements required for activation. Consequently, this prevents the receptor from transitioning to the active state or returning to the inactive state, effectively trapping it in an intermediate configuration.

A key implication of these structural observations is that α-CT displacement at the primary insulin-binding site can generate antagonistic signaling outcomes by locking the receptor between inactive and active states. Thus, fusion geometry, not merely binding affinity, determines whether a synthetic ligand stabilizes an active, biased, or antagonistic configuration, consistent with recent studies using de novo protein design^61^. Hybrid ligands incorporating site-2 elements into insulin (e.g., fusions at LysB29 or the C-terminal A-chain) further demonstrate that the modular assembly of binding motifs can convert insulin into partial agonists or antagonists^62,63^. Collectively, these studies establish that IR activation can be synthetically programmed through ligand topology.

### Computational protein design

Defining how extracellular conformational changes are transmitted to intracellular signaling and trafficking has been challenging because of the structural complexity of IR–ligand assemblies. Computational protein design has recently enabled direct interrogation of this structure–function relationship^61,64^.

De novo designed IR binders were generated against individual extracellular domains and fused in defined orientations with tunable linker flexibility^61^. The site-1 (S1) binder replaces the α-CT interaction at the L1 domain, whereas the site-2 (S2) binder engages the FnIII-1 domain (Fig. 2c, d). Each module is ~7 kDa, cysteine-free, thermostable, and readily produced. Remarkably, reversing the fusion topology produced opposite functional outcomes: S2–S1 constructs act as agonists, whereas S1–S2 constructs function as antagonists. Mechanistically, designer agonists must coordinate simultaneous engagement of two receptor domains through a covalent fusion^61^. In S1–S2 constructs, although spatial proximity in the apo-IR may allow dual binding, the extended linker required to accommodate conformational transitions likely prevents stabilization of the active T-shaped conformation, explaining their antagonistic behavior, similar to that observed for S661.

By contrast, S2–S1 constructs appear to operate via a stepwise activation mechanism. Because the S2 epitope on FnIII-1 is exposed in the apo state, S2 likely binds first, inducing partial receptor opening and bringing the L1 and FnIII-1 into closer proximity. The S1 module can then engage L1, stabilizing the active conformation (Fig. 2c,d). As in the S597-bound structure, two S2–S1 constructs bind to the IR and stabilize the extended T-shaped active conformation. Linker rigidity further tunes signaling output: more rigid constructs (e.g., RF-405 and RF-409) favor compact, stable active geometries and function as balanced agonists, activating both metabolic and mitogenic pathways (Fig. 2d). Conversely, flexible constructs (e.g., S2-F1-S1) act as biased agonists, preferentially activating metabolic signaling. Rigid constructs induce IR autophosphorylation comparable with insulin and efficiently promote downstream signaling and receptor trafficking, whereas flexible constructs induce partial autophosphorylation, particularly reduced in the C-terminal region, resulting in attenuated mitogenic signaling and altered trafficking dynamics^61,65^ (Fig. 2c,d).

In vivo, these designer agonists potently lower glucose levels with slower onset but a more prolonged duration than insulin. Because they engage IR through a non-canonical geometry, they can bypass certain disease-causing IR mutations. Moreover, they display high selectivity for IR and reduced stimulation of cancer cell proliferation compared with insulin.

Collectively, computationally designed IR ligands demonstrate that receptor activation is governed by binding topology, linker mechanics, and nanoscale geometry. These findings provide direct evidence that extracellular conformational programming can be leveraged to tune signaling bias, trafficking behavior, and therapeutic output.

### Aptamer-based insulin receptor modulators

DNA aptamers represent a distinct class of IR modulators capable of stabilizing specific conformational states.

#### IR-A43: cooperative potentiator

IR-A43 was identified as a modulator that does not activate IR independently but enhances insulin-dependent signaling^66^. IR-A43 and insulin bind to the receptor with mutual positive cooperativity (Fig. 2e). Alone, IR-A43 is largely inactive; however, in the presence of insulin, it potentiates IR activation. The overall structure of IR with IR-A43 and insulin is nearly identical to the single-insulin-bound state (gamma shape)^67^. Importantly, IR-A43 does not displace the α-CT motif from the L1 domain. Instead, IR-A43 binds to the site formed by the CR, L2, and FnIII-1 domains, corresponding to site-2 on the protomer opposite the primary insulin-binding site. IR-A43 potentiates insulin signaling by stabilizing this asymmetric conformation while limiting progression to second insulin binding. Thus, IR-A43 enhances signaling by favoring a specific occupancy state rather than directly activating the receptor.

#### IR-A62: context-dependent allosteric modulator

IR-A62 exhibits concentration-dependent regulatory behavior^67^. IR-A62 preferentially enhances monophosphorylation at Y1150 and can function as a positive or negative allosteric modulator depending on its concentration. Unlike IR-A43, IR-A62 alone activates IR. At low concentrations in the presence of insulin, IR-A62 enhances insulin binding and potentiates receptor activation. At higher concentrations, however, it competes with insulin and behaves as a negative modulator. Consistent with this, in vivo, IR-A62 demonstrated prolonged glucose-lowering effects in db/db mice compared with insulin and was capable of reducing glucose levels in streptozotocin-treated mice even in the absence of insulin, indicating partial agonist activity under insulin-deficient conditions.

Structural analyses provide insight into these functional properties^67^. In the absence of insulin, two IR-A62 aptamers bind symmetrically to the IR dimer, displacing the α-CT from the L1 domain and directly engaging L1. This binding stabilizes a distinct “arrowhead”-shaped receptor conformation. In the presence of insulin, IR-A62 binds asymmetrically, engaging the opposite site-1/site-2′ interface and effectively bridging protomers (Fig. 2f). In this configuration, IR-A62 appears to trap receptor intermediates by mimicking insulin binding at site-1/site-2′ hybrid site^50^.

### Antibody-based modulators of insulin receptor

Monoclonal antibodies targeting IR represent the earliest non-insulin ligands shown to modulate receptor activity^68^. Depending on epitope location and binding geometry, these antibodies function as partial agonists, positive allosteric modulators, or antagonists, providing key mechanistic insights into IR activation beyond canonical insulin binding.

#### Anti-IR monoclonal antibodies

Earlier monoclonal antibodies, including 83-7, 83-14, 18-44, and 18-146, were generated by immunizing mice with IM-9 lymphocytes or purified placental receptor and were extensively characterized biochemically^69,70^. Among them, mAb 83-7 and 83-14 were widely used for the structural determination of IR and for functional studies^17^. mAb 83-7, which binds within the CR domain (residues 233–281), modestly enhances insulin binding without affecting dissociation and stimulates kinase activation, glucose uptake, and lipogenesis^71^. mAb 83-14, mapping to the FnIII-1 domain near the site-2 surface, inhibits insulin site-2 binding and dissociation, yet paradoxically stimulates receptor kinase activity and metabolic signaling. Because mAb 83-14 binds to the site-2 surface rather than the primary binding site, it synergistically enhances IR activation in the presence of insulin, suggesting that mAb 83-14 may promote conformational changes that favor an optimally active state of IR.

mAb m83-7 and 83-14 can activate IR and preferentially stimulate AKT phosphorylation with minimal ERK activation, indicating partial and biased agonism^71^. In general, antibody-induced IR autophosphorylation is lower than that achieved with insulin or S597. A defining feature of antibody ligands is their size and bivalency. Antibodies can cross-link receptors at the cell surface, potentially inducing higher-order clustering or enforcing specific ectodomain geometries. Antibody stimulation is frequently associated with pronounced receptor internalization and downregulation^72^, suggesting that altered trafficking dynamics contribute to their signaling profile. The large molecular size of antibodies may also restrict receptor flexibility, limiting reversion to the apo conformation and thereby stabilizing an active or inactive state depending on epitope engagement.

#### XMet series: engineered allosteric modulators

The XMet antibodies (XMetA, XMetS, and XMetD) represent a well-characterized class of IR-modulating antibodies with distinct pharmacological properties. XMetA functions as a partial agonist and can directly activate the IR in the absence of insulin^73–75^. It stimulates receptor autophosphorylation and promotes glucose uptake in vitro, with preferential activation of AKT relative to ERK. In vivo, XMetA lowers blood glucose and improves metabolic parameters in diabetic rodent models and diabetic cynomolgus monkeys. These findings suggest that XMetA acts as a positive allosteric activator of the IR with reduced mitogenic signaling compared with insulin. Although the precise epitope remains incompletely defined, its bivalent nature suggests that receptor cross-linking or stabilization of an active ectodomain conformation underlies its activity.

XMetS does not directly activate IR but enhances insulin binding and potentiates insulin-stimulated signaling^76^. In diet-induced obese mice, XMetS improves glucose tolerance and reduces compensatory hyperinsulinemia. Accordingly, XMetS is classified as a positive allosteric modulator. XMetS may stabilize the insulin-bound state or facilitate conformational transitions toward the active receptor configuration, although structural validation remains lacking.

By contrast, XMetD functions as a negative modulator^77^. It reduces insulin binding, attenuates insulin-dependent IR activation, and reverses hypoglycemia in hyperinsulinemic mouse models. XMetD binds both insulin receptor isoform A (IR-A) and insulin receptor isoform B (IR-B) and likely interferes with insulin engagement through an allosteric mechanism. Collectively, the XMet series illustrates how epitope selection and antibody geometry can tune IR signaling from activation to inhibition, even in the absence of detailed structural information.

### Nano-insulin and receptor clustering

Super-resolution imaging studies reveal that IR exists as preformed dimers organized into nanoclusters at the plasma membrane, with an average diameter of ~74 nm (approximately 3–4 IR dimers per cluster)^45^. Insulin stimulation modestly increases cluster size (~84 nm) but does not fundamentally reorganize this architecture, indicating that IR signaling occurs within a pre-existing nanoscale framework. To directly determine how ligand geometry influences activation, a DNA origami-based “NanoRod” platform (~140 nm in length) was engineered to present insulin with defined valency and spacing. Increasing the insulin valency enhanced the IR binding affinity and markedly reduced the dissociation rates, indicating prolonged receptor residency. Multivalent insulin NanoRods induced stronger IR autophosphorylation and downstream transcriptional responses than monovalent insulin, demonstrating that ligand valency and spatial arrangement directly modulate signaling strength. These findings highlight that IR activation is sensitive to nanoscale ligand architecture, not solely ligand identity. Whether multivalent insulin alters signaling bias or receptor trafficking remains to be determined.

## Disease contexts of non-canonical insulin receptor signaling

### Diabetes

**Fig. 3:**
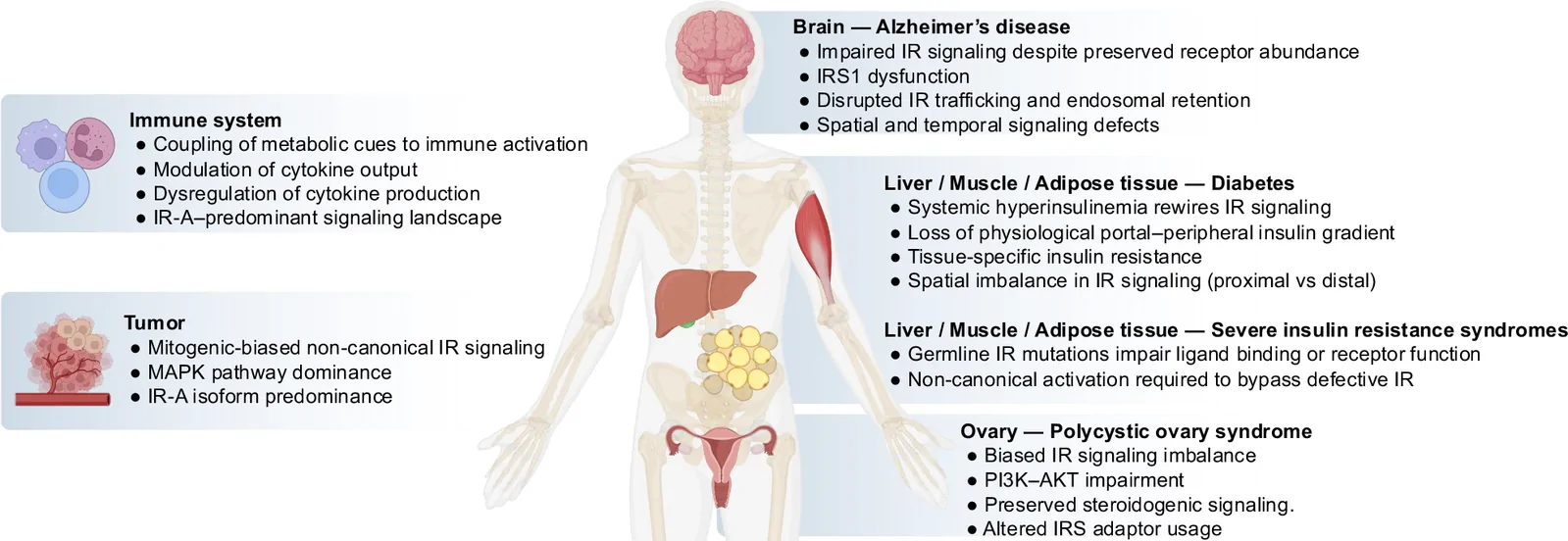
Disease-specific distortion of insulin receptor signaling. Across diverse diseases, IR signaling is qualitatively rewired through changes in pathway balance, adaptor usage, receptor trafficking, and insulin dynamics, resulting in context-dependent signaling outputs in metabolic, neural, immune, and proliferative tissues.

Insulin therapy is the primary treatment for type 1 diabetes and is frequently used in type 2 diabetes to control blood glucose levels (Fig. 3). Engineered insulin analogs, including rapid-acting, long-acting, and glucose-responsive insulins, have expanded therapeutic options for diabetes management, yet important limitations persist^78–81^. Although newly designed insulin mimetics can activate insulin signaling independently of insulin, they are unlikely to replace the current insulin therapy for diabetes. Instead, one potential advantage of de novo designed agonists lies in their high receptor selectivity and the possibility of achieving tissue-specific targeting.

Under physiological conditions, insulin secreted from the pancreas first enters the portal circulation and is delivered predominantly to the liver, establishing a gradient from the liver to peripheral tissues^82,83^. Conversely, systemic insulin administration disrupts this physiological gradient, which may not fully recapitulate native insulin action and may contribute to selective hepatic insulin resistance. Selective hepatic insulin resistance in type 2 diabetes has important downstream metabolic consequences^84^. In hepatic insulin resistance, impaired suppression of gluconeogenesis and hepatic glucose production contributes to fasting hyperglycemia, whereas lipogenic signaling can remain preserved, promoting de novo lipogenesis, hypertriglyceridemia, and hepatic lipid accumulation. This dissociation is clinically important because it helps explain why patients with type 2 diabetes often exhibit both hyperglycemia and atherogenic dyslipidemia despite elevated circulating insulin levels.

Because insulin is a small peptide in which many residues are tightly constrained by receptor-binding requirements^17,19^, opportunities for engineering insulin itself are relatively limited. By contrast, newly developed ligands are often larger and more modular, allowing modifications that can alter receptor-binding geometry, ligand valency, and tissue delivery properties. Such engineering strategies may enable the development of next-generation insulin mimetics with improved therapeutic profiles for diabetes.

### Severe insulin resistance syndromes

Rare germline mutations in IR provide the most direct evidence that intact IR signaling is indispensable for human physiology. Loss-of-function mutations in IR cause severe insulin resistance syndromes such as Donohue syndrome and Rabson–Mendenhall syndrome, which are marked by extreme hyperinsulinemia, profound growth retardation, multisystem metabolic dysregulation, and early mortality^85^ (Fig. 3).

Many mutations occur within insulin-binding sites or kinase domains, whereas others impair receptor processing and reduce the abundance of functional receptors at the cell surface. Current therapeutic options, such as supraphysiological insulin or IGF1 treatment, provide only temporary benefits, but effective long-term therapeutic strategies remain limited^86–89^. Given the diverse functions of IR in metabolic and non-metabolic tissues, gene therapy approaches remain challenging. Recent advances in insulin mimetics that activate IR through mechanisms distinct from insulin offer potential therapeutic opportunities for these rare diseases, particularly for mutations that impair insulin binding or disrupt insulin-dependent receptor activation^57^. Because these disorders arise early in life and are associated with severe developmental abnormalities, therapeutic strategies that restore both metabolic and mitogenic signaling, rather than selectively biased signaling, may be particularly beneficial for these conditions.

### Cancer

Epidemiological studies have long noted associations between hyperinsulinemia, obesity, and increased cancer incidence or progression, particularly in colorectal, pancreatic, breast, endometrial, ovarian and liver cancers, suggesting that IR signaling can act as a permissive factor for tumor growth in metabolically dysregulated states (Fig. 3). Importantly, this relationship highlights the ability of IR signaling to actively support tumor growth and progression in metabolically permissive contexts^10,90,91^.

At the cellular level, many cancers exhibit altered IR signaling landscapes, including increased IR expression and preferential use of mitogenic downstream pathways. Insulin can directly promote tumor growth in vivo under defined conditions^92–95^. In xenograft and allograft models, systemic insulin administration or hyperinsulinemic states enhance tumor growth and proliferation. In particular, cancer-associated rewiring of IR signaling favors mitogenic outputs over metabolic responses through altered adaptor utilization and MAPK pathway engagement. In addition, epidemiological studies indicate that hyperinsulinemia is associated with increased cancer risk, progression, and mortality, supporting a role for sustained insulin exposure in promoting tumor growth^96,97^.

The pathological consequences of mitogenic bias are strongly supported by studies of engineered insulin analogs with enhanced growth-promoting activity. The super-mitogenic insulin analog Asp(B10) exhibits disproportionately high mitogenic potency relative to its metabolic activity, induces mammary tumors in rodents, and is widely used as a positive control for carcinogenic risk assessment^98–100^. Cancer-associated bias in IR signaling is further reinforced by differential expression of IR isoforms. Many tumors preferentially express the IR-A isoform, which lacks exon 11 and exhibits high affinity for insulin-like growth factor 2 (IGF2). Structural studies of IGF2 binding to IR have further revealed how exon 11-dependent differences in the α-CT region impose distinct constraints on ligand engagement by IR-A and IR-B^101^. IGF2 binding to IR-A promotes sustained receptor activation and mitogenic signaling, establishing an autocrine IGF2–IR-A loop that supports tumor growth and survival^102–104^.

### Polycystic ovary syndrome

Polycystic ovary syndrome (PCOS) is strongly associated with insulin resistance and compensatory hyperinsulinemia that are not solely explained by obesity, placing dysregulated IR signaling at the center of its pathophysiology^105–108^ (Fig. 3). Clinical and experimental studies show that impaired insulin action contributes to not only metabolic abnormalities but also reproductive dysfunction, including ovarian hyperandrogenism and anovulation. Hyperinsulinemia amplifies ovarian androgen production both directly, by acting on theca cells, and indirectly, by suppressing hepatic sex hormone-binding globulin (SHBG) production, thereby increasing circulating free androgen levels and enhancing androgen bioavailability, which exacerbates endocrine imbalance^107^.

At the molecular level, insulin resistance in PCOS exhibits features of selective insulin resistance, characterized by impaired metabolic signaling with relative preservation of growth-related pathways^109^. Studies in skeletal muscle from women with PCOS demonstrate marked reductions in insulin-stimulated IRS1-associated PI3K activity despite relatively normal IR expression and autophosphorylation, indicating a post-receptor defect centered on the IRS–PI3K–AKT metabolic axis^110^. Alterations in adaptor protein usage may contribute to this signaling imbalance. Skeletal muscle from individuals with PCOS exhibits increased IRS2 expression in the setting of reduced IRS1-dependent signaling, suggesting compensatory shifts in adaptor utilization^111^. In the ovarian tissue, altered patterns of IRS family member expression have also been reported in a cell-type-specific manner. Several studies describe reduced IRS1 immunoreactivity in granulosa cells together with increased IRS2 expression in theca or follicular compartments compared with ovaries from control individuals without PCOS^112,113^; however, other analyses report coordinated upregulation of IRS1 and IRS2 specifically in theca cells^114^. These changes in IRS protein abundance and localization are associated with altered insulin responsiveness and can be modulated by insulin sensitizers such as troglitazone, supporting that changes in adaptor remodeling contribute to tissue-specific IR signaling alterations that promote androgen excess in PCOS ovaries^113,114^.

Taken together, PCOS represents a disease state characterized by imbalanced IR signaling in which metabolic responses are selectively impaired. This suggests that therapeutic strategies capable of reinforcing metabolic IR signaling may be beneficial in PCOS, although this concept remains to be directly tested, including through studies examining the effects of insulin and insulin mimetics on ovarian steroidogenic signaling.

### Immune and inflammatory responses

IR signaling plays an important regulatory role in immune and inflammatory responses by influencing immune cell metabolism, survival, and functional activation across both innate and adaptive immune compartments (Fig. 3). IR signaling modulates immune cell bioenergetic capacity and cytokine production, thereby coupling systemic metabolic cues to immune function^8,115^. Multiple immune cell populations, including lymphocytes and macrophages, express IR, allowing insulin to exert direct cell-intrinsic effects on immune responses^8^.

In innate immune cells, insulin-responsive pathways influence macrophage activation states and inflammatory cytokine output, contributing to regulation of inflammatory tone^116^. In adaptive immunity, T cell-specific deletion of IR results in impaired clonal expansion, reduced effector cytokine production, and defective pathogen clearance, indicating that insulin-dependent metabolic support is required for effective T cell-mediated host defense^117^. IR signaling can also modulate chronic inflammatory disease processes. For example, insulin enhances the expression of proinflammatory cytokines and chemokines in periodontal ligament cells and gingival fibroblasts under hyperinsulinemia conditions^11^.

Immune IR signaling is further shaped by tissue-dependent expression of IR isoforms^118,119^. The IR-B isoform predominates in classical metabolic tissues such as liver, skeletal muscle, and adipose tissue, whereas IR-A is preferentially expressed in non-metabolic tissues including spleen and hematopoietic compartments. IR-A exhibits broader ligand responsiveness, including high-affinity binding to IGF2, whereas IR-B is more selective for insulin and optimized for metabolic signaling^118^. Comparative proteomic analysis demonstrates that IR-A preferentially engages signaling networks associated with immune and growth-related signaling, whereas IR-B signaling is enriched for metabolic regulatory programs^119^. These findings suggest that future insulin mimetics could exploit these structural features to selectively modulate IR isoform-specific signaling.

### Alzheimer disease

IR signaling in the central nervous system (CNS) regulates neuronal survival, energy metabolism, gene expression, and synaptic maintenance (Fig. 3). In particular, IR signaling in hippocampal and cortical regions is selectively vulnerable in AD^120,121^. The disruption of neuronal IR signaling has been linked to alterations in synaptic function, cerebral bioenergetics, and cognitive performance^121^. Experimental studies further demonstrate that IR signaling directly regulates synapse density, dendritic structural plasticity, and circuit-level function, indicating its role in the maintenance of synaptic connectivity^122^.

The analyses of postmortem human brain tissue demonstrate that AD is associated with intrinsic brain insulin resistance^123^. Ex vivo stimulation of hippocampal tissue reveals markedly reduced insulin responses in the AKT pathway despite largely preserved levels of signaling proteins^123^. These impairments are most prominent at the level of IRS1 and are consistently associated with increased inhibitory IRS1 serine phosphorylation. Biomarkers of IRS1 serine phosphorylation increase progressively from cognitively normalcy to mild cognitive impairment and AD and correlate negatively with memory performance^123^. Importantly, total levels of IR, IRS1, AKT, GSK3β, mTOR, and ERK remain largely preserved^123^, indicating that impaired IR signaling in AD primarily reflects altered activation states rather than loss of signaling components.

Amyloid-β (Aβ) oligomers can directly disrupt neuronal IR signaling^124^. Exposure to Aβ oligomers activates stress pathways, including JNK signaling, leading to increased inhibitory serine phosphorylation and reduced tyrosine phosphorylation of IRS1. These alterations impair insulin signal propagation and are associated with synaptic dysfunction.

Apolipoprotein E4 (APOE4), a strong genetic risk factor for AD, has been shown to impair neuronal IR signaling by disrupting IR trafficking^125^. In mice where the murine *Apoe* locus is replaced with the human *APOE4* allele under the control of endogenous regulatory elements, insulin-stimulated activation of AKT and GSK3β is reduced. Mechanistically, APOE4 binds IR, interferes with insulin–IR interaction, and promotes retention of IR within endosomal compartments, thereby reducing receptor availability at the cell surface and impairing downstream signal propagation.

Consistent with this, IR signaling in neurons is subject to spatial regulation. Live-cell imaging studies demonstrate dynamic lateral mobility and compartment-specific confinement of IR across somatodendritic membranes, and receptor cycling is thought to influence downstream signaling^126^. Together, these observations support the idea that disruption of adaptor phosphorylation, receptor trafficking, and spatial organization contribute to impaired IR signaling in AD.

## Future directions

Advances in protein engineering and computational design provide new opportunities to interrogate and manipulate IR signaling. Engineered ligands can stabilize distinct receptor conformations and selectively bias downstream signaling. These tools not only enable mechanistic dissection of IR signaling but also offer a foundation for developing next-generation insulin mimetics with improved safety and functional specificity.

A key future direction is achieving cell-type-specific and compartment-specific control of IR signaling. Because signaling outputs vary across tissues and intracellular locations, strategies that direct receptor engagement to defined cellular context may allow more precise therapeutic modulation. Approaches combining ligand engineering with targeted delivery or nanoscale ligand presentation may enable selective activation of IR signaling in metabolically relevant tissues while minimizing unintended effects in other organs.

These advances have important implications for precision medicine. Many diseases linked to IR signaling likely involve qualitative rewiring of signaling rather than simple changes in receptor activity. Therapeutic strategies that selectively adjust signaling balance may therefore provide more effective and context-specific interventions than approaches that globally increase IR signaling.

However, several key challenges remain. It is still unclear which features define beneficial versus pathological signaling bias, how signaling bias can be maintained in complex physiological environments, and how receptor trafficking, ligand geometry, and adaptor selection are integrated in vivo. In addition, significant translational challenges must also be addressed before engineered IR ligands can be broadly applied therapeutically (Table 1). Different classes of insulin mimetics possess distinct pharmacological advantages and limitations. Peptides and aptamers generally offer favorable manufacturability but often require strategies to improve stability, circulation half-life, and route of administration. Antibodies benefit from prolonged systemic exposure but may display limited tissue penetration, whereas de novo designed proteins may face additional challenges related to pharmacokinetic optimization and potential immunogenicity. In addition, tissue accessibility represents an important consideration for disease-specific applications. For example, therapeutic modulation of neuronal IR signaling in AD will likely require strategies capable of achieving sufficient CNS exposure.

Encouragingly, recent advances in GLP1 receptor agonist development demonstrate how molecular engineering approaches, including fatty acid conjugation, albumin binding, Fc fusion, and sustained-release delivery systems, can dramatically improve peptide stability, pharmacokinetic properties, and tissue accessibility^127^. Notably, several GLP1 receptor agonists exert central effects on appetite regulation, indicating that engineered peptide therapeutics can access relevant CNS targets. These advances provide a useful framework for the future development of peptide-based IR modulators. Addressing these translational challenges will be essential for realizing the therapeutic potential of next-generation IR modulators.

Taken together, these developments suggest that insulin action can be understood not simply as a consequence of receptor activation, but as a programmable signaling network. Defining and controlling the qualitative features of IR signaling may enable a shift from insulin replacement toward precise reprogramming of receptor function in human disease.

## References

[1] Banting, F. G. & Best, C. H. The internal secretion of the pancreas. 1922. Indian J. Med Res 125, 251–266 (2007). 17582843

[2] Banting, F. G., Best, C. H., Collip, J. B., Campbell, W. R. & Fletcher, A. A. Pancreatic extracts in the treatment of diabetes mellitus. Can. Med Assoc. J. 12, 141–146 (1922). PMC1524425 20314060

[3] Cuatrecasas, P., Desbuquois, B. & Krug, F. Insulin-receptor interactions in liver cell membranes. *Biochem Biophys. Res Commun.* **44**, 333–339 10.1016/0006-291x(71)90604-8 (1971). 4334135

[4] Ebina, Y. et al. The human insulin receptor cDNA: the structural basis for hormone-activated transmembrane signalling. *Cell* **40**, 747–758 10.1016/0092-8674(85)90334-4 (1985). 2859121

[5] Kasuga, M., Karlsson, F. A. & Kahn, C. R. Insulin stimulates the phosphorylation of the 95,000-dalton subunit of its own receptor. *Science* **215**, 185–187 10.1126/science.7031900 (1982). 7031900

[6] Boucher, J., Kleinridders, A. & Kahn, C. R. Insulin receptor signaling in normal and insulin-resistant states. Cold Spring Harb. Perspect. Biol. 6, a009191 10.1101/cshperspect.a009191 (2014). PMC3941218 24384568

[7] Pomytkin, I. et al. Insulin receptor in the brain: mechanisms of activation and the role in the CNS pathology and treatment. *CNS Neurosci. Ther.* **24**, 763–774 10.1111/cns.12866 (2018). PMC6489906 29691988

[8] Makhijani, P. et al. Regulation of the immune system by the insulin receptor in health and disease. *Front Endocrinol. (Lausanne)* **14**, 1128622 10.3389/fendo.2023.1128622 (2023). PMC10040865 36992811

[9] Hale, L. J. & Coward, R. J. Insulin signalling to the kidney in health and disease. *Clin. Sci. (Lond.)* **124**, 351–370 10.1042/CS20120378 (2013). 23190266

[10] Gallagher, E. J. & LeRoith, D. Hyperinsulinaemia in cancer. *Nat. Rev. Cancer* **20**, 629–644 10.1038/s41568-020-0295-5 (2020). 32908223

[11] Sohn, Y., Jeong, H.J. & Park, J. The Link between Hyperinsulinemia and Periodontitis in Diabetics. J. Dent. Res 105(3), 294–301 10.1177/00220345251366952 (2026). 41108158

[12] Spoto, B., Pisano, A. & Zoccali, C. Insulin resistance in chronic kidney disease: a systematic review. *Am. J. Physiol. Ren. Physiol.* **311**, F1087–F1108 10.1152/ajprenal.00340.2016 (2016). 27707707

[13] Dall’Agnese, A. et al. The dynamic clustering of insulin receptor underlies its signaling and is disrupted in insulin resistance. *Nat. Commun.* **13**, 7522 10.1038/s41467-022-35176-7 (2022). PMC9727033 36473871

[14] Hall, C., Yu, H. & Choi, E. Insulin receptor endocytosis in the pathophysiology of insulin resistance. *Exp. Mol. Med* **52**, 911–920 10.1038/s12276-020-0456-3 (2020). PMC7338473 32576931

[15] Ward, C. W. & Lawrence, M. C. Ligand-induced activation of the insulin receptor: a multi-step process involving structural changes in both the ligand and the receptor. *Bioessays* **31**, 422–434 10.1002/bies.200800210 (2009). 19274663

[16] Croll, T. I. et al. Higher-resolution structure of the human insulin receptor ectodomain: multi-modal inclusion of the insert domain. *Structure* **24**, 469–476 10.1016/j.str.2015.12.014 (2016). PMC4860004 26853939

[17] McKern, N. M. et al. Structure of the insulin receptor ectodomain reveals a folded-over conformation. *Nature* **443**, 218–221 10.1038/nature05106 (2006). 16957736

[18] Scapin, G. et al. Structure of the insulin receptor-insulin complex by single-particle cryo-EM analysis. *Nature* **556**, 122–125 10.1038/nature26153 (2018). PMC5886813 29512653

[19] Uchikawa, E., Choi, E., Shang, G., Yu, H. & Bai, X. C. Activation mechanism of the insulin receptor revealed by cryo-EM structure of the fully liganded receptor-ligand complex. Elife 8, e48630 10.7554/eLife.48630 (2019). PMC6721835 31436533

[20] Menting, J. G. et al. How insulin engages its primary binding site on the insulin receptor. *Nature* **493**, 241–245 10.1038/nature11781 (2013). PMC3793637 23302862

[21] Choi, E. & Bai, X. C. The activation mechanism of the insulin receptor: a structural perspective. *Annu Rev. Biochem* **92**, 247–272 10.1146/annurev-biochem-052521-033250 (2023). PMC10398885 37001136

[22] Choi, E., Duan, C. & Bai, X. C. Regulation and function of insulin and insulin-like growth factor receptor signalling. *Nat. Rev. Mol. Cell Biol.* **26**, 558–580 10.1038/s41580-025-00826-3 (2025). PMC12631569 39930003

[23] White, M. F., Shoelson, S. E., Keutmann, H. & Kahn, C. R. A cascade of tyrosine autophosphorylation in the beta-subunit activates the phosphotransferase of the insulin receptor. *J. Biol. Chem.* **263**, 2969–2980 (1988). 2449432

[24] Hubbard, S. R. Crystal structure of the activated insulin receptor tyrosine kinase in complex with peptide substrate and ATP analog. *EMBO J.* **16**, 5572–5581 10.1093/emboj/16.18.5572 (1997). PMC1170189 9312016

[25] Danielsen, A. G. & Roth, R. A. Role of the juxtamembrane tyrosine in insulin receptor-mediated tyrosine phosphorylation of p60 endogenous substrates. *Endocrinology* **137**, 5326–5331 10.1210/endo.137.12.8940353 (1996). 8940353

[26] White, M. F. et al. Mutation of the insulin receptor at tyrosine 960 inhibits signal transmission but does not affect its tyrosine kinase activity. *Cell* **54**, 641–649 10.1016/s0092-8674(88)80008-4 (1988). 2842060

[27] Sun, X. J. et al. Structure of the insulin receptor substrate IRS-1 defines a unique signal transduction protein. *Nature* **352**, 73–77 10.1038/352073a0 (1991). 1648180

[28] White, M. F. & Kahn, C. R. The insulin signaling system. *J. Biol. Chem.* **269**, 1–4 (1994). 8276779

[29] Myers, M. G. Jr. et al. IRS-1 activates phosphatidylinositol 3’-kinase by associating with src homology 2 domains of p85. *Proc. Natl. Acad. Sci. USA.* **89**, 10350–10354 10.1073/pnas.89.21.10350 (1992). PMC50336 1332046

[30] Pronk, G. J., McGlade, J., Pelicci, G., Pawson, T. & Bos, J. L. Insulin-induced phosphorylation of the 46- and 52-kDa Shc proteins. *J. Biol. Chem.* **268**, 5748–5753 (1993). 8449939

[31] Sasaoka, T. et al. Evidence for a functional role of Shc proteins in mitogenic signaling induced by insulin, insulin-like growth factor-1, and epidermal growth factor. *J. Biol. Chem.* **269**, 13689–13694 (1994). 7513704

[32] Sasaoka, T., Draznin, B., Leitner, J. W., Langlois, W. J. & Olefsky, J. M. Shc is the predominant signaling molecule coupling insulin receptors to activation of guanine nucleotide releasing factor and p21ras-GTP formation. *J. Biol. Chem.* **269**, 10734–10738 (1994). 8144662

[33] Yamauchi, K. & Pessin, J. E. Insulin receptor substrate-1 (IRS1) and Shc compete for a limited pool of Grb2 in mediating insulin downstream signaling. *J. Biol. Chem.* **269**, 31107–31114 (1994). 7983051

[34] Hausmann, S. & Ussar, S. Insulin receptor trafficking steers insulin action. *Mol. Metab.* **5**, 253–254 10.1016/j.molmet.2016.02.004 (2016). PMC4811990 27069864

[35] Murphy, J. E., Padilla, B. E., Hasdemir, B., Cottrell, G. S. & Bunnett, N. W. Endosomes: a legitimate platform for the signaling train. *Proc. Natl. Acad. Sci. USA* **106**, 17615–17622 10.1073/pnas.0906541106 (2009). PMC2764915 19822761

[36] Kjaergaard, J. et al. Personalized molecular signatures of insulin resistance and type 2 diabetes. *Cell* **188**, 4106–4122.e4116 10.1016/j.cell.2025.05.005 (2025). 40436015

[37] Walz, H. A. et al. Isoform-specific regulation of Akt signaling by the endosomal protein WDFY2. *J. Biol. Chem.* **285**, 14101–14108 10.1074/jbc.M110.110536 (2010). PMC2863185 20189988

[38] Park, J. et al. MAD2-dependent insulin receptor endocytosis regulates metabolic homeostasis. *Diabetes* **72**, 1781–1794 10.2337/db23-0314 (2023). PMC10658066 37725942

[39] Choi, E., Zhang, X., Xing, C. & Yu, H. Mitotic checkpoint regulators control insulin signaling and metabolic homeostasis. *Cell* **166**, 567–581 10.1016/j.cell.2016.05.074 (2016). PMC5560052 27374329

[40] Liu, X. et al. Insulin induces insulin receptor degradation in the liver through EphB4. *Nat. Metab.* **4**, 1202–1213 10.1038/s42255-022-00634-5 (2022). 36131205

[41] Ansarullah et al. Inceptor counteracts insulin signalling in beta-cells to control glycaemia. *Nature* **590**, 326–331 10.1038/s41586-021-03225-8 (2021). 33505018

[42] Gustavsson, J. et al. Localization of the insulin receptor in caveolae of adipocyte plasma membrane. *FASEB J.* **13**, 1961–1971 (1999). 10544179

[43] Jarett, L. & Smith, R. M. The natural occurrence of insulin receptors in groups on adipocyte plasma membranes as demonstrated with monomeric ferritin-insulin. *J. Supramol. Struct.* **6**, 45–59 10.1002/jss.400060104 (1977). 895138

[44] Li, H. et al. Mechanism of INSR clustering with insulin activation and resistance revealed by super-resolution imaging. *Nanoscale* **14**, 7747–7755 10.1039/d2nr01051h (2022). 35579582

[45] Spratt, J. et al. Multivalent insulin receptor activation using insulin-DNA origami nanostructures. *Nat. Nanotechnol.* **19**, 237–245 10.1038/s41565-023-01507-y (2024). PMC10873203 37813939

[46] Jiang, Z. Y. et al. Characterization of selective resistance to insulin signaling in the vasculature of obese Zucker (fa/fa) rats. *J. Clin. Invest* **104**, 447–457 10.1172/JCI5971 (1999). PMC408521 10449437

[47] Kubota, H. et al. Temporal coding of insulin action through multiplexing of the AKT pathway. *Mol. Cell* **46**, 820–832 10.1016/j.molcel.2012.04.018 (2012). 22633957

[48] Matveyenko, A. V. et al. Pulsatile portal vein insulin delivery enhances hepatic insulin action and signaling. *Diabetes* **61**, 2269–2279 10.2337/db11-1462 (2012). PMC3425431 22688333

[49] Nielsen, J. et al. Structural investigations of full-length insulin receptor dynamics and signalling. *J. Mol. Biol.* **434**, 167458 10.1016/j.jmb.2022.167458 (2022). 35074483

[50] Li, J. et al. Synergistic activation of the insulin receptor via two distinct sites. *Nat. Struct. Mol. Biol.* **29**, 357–368 10.1038/s41594-022-00750-6 (2022). PMC9115778 35361965

[51] Brandt, S. J., Mayer, J. P., Ford, J., Gelfanov, V. M. & DiMarchi, R. D. Controlled intramolecular antagonism as a regulator of insulin receptor maximal activity. *Peptides* **100**, 18–23 10.1016/j.peptides.2017.11.022 (2018). 29412818

[52] Jensen, M., Hansen, B., De Meyts, P., Schaffer, L. & Urso, B. Activation of the insulin receptor by insulin and a synthetic peptide leads to divergent metabolic and mitogenic signaling and responses. *J. Biol. Chem.* **282**, 35179–35186 10.1074/jbc.M704599200 (2007). 17925406

[53] Pillutla, R. C. et al. Peptides identify the critical hotspots involved in the biological activation of the insulin receptor. *J. Biol. Chem.* **277**, 22590–22594 10.1074/jbc.M202119200 (2002). 11964401

[54] Schaffer, L. et al. Assembly of high-affinity insulin receptor agonists and antagonists from peptide building blocks. *Proc. Natl. Acad. Sci. USA* **100**, 4435–4439 10.1073/pnas.0830026100 (2003). PMC153573 12684539

[55] Frikke-Schmidt, H. et al. Treatment of diabetic rats with insulin or a synthetic insulin receptor agonist peptide leads to divergent metabolic responses. *Diabetes* **64**, 1057–1066 10.2337/db14-0914 (2015). 25315006

[56] Kanter, J. E. et al. A novel strategy to prevent advanced atherosclerosis and lower blood glucose in a mouse model of metabolic syndrome. *Diabetes* **67**, 946–959 10.2337/db17-0744 (2018). PMC5909997 29483182

[57] Park, J. et al. Activation of the insulin receptor by an insulin mimetic peptide. *Nat. Commun.* **13**, 5594 10.1038/s41467-022-33274-0 (2022). PMC9508239 36151101

[58] Lawrence, C. F. et al. Insulin mimetic peptide disrupts the primary binding site of the insulin receptor. *J. Biol. Chem.* **291**, 15473–15481 10.1074/jbc.M116.732180 (2016). PMC4957034 27281820

[59] Schaffer, L. et al. A novel high-affinity peptide antagonist to the insulin receptor. *Biochem Biophys. Res Commun.* **376**, 380–383 10.1016/j.bbrc.2008.08.151 (2008). 18782558

[60] Polák, M. et al. Antagonistic insulin mimetics lock the insulin receptor in an alternative apo-state. Preprint at *bioRxiv* 10.1101/2025.08.05.667158 (2025).

[61] Wang, X. et al. Tuning insulin receptor signaling using de novo-designed agonists. *Mol. Cell* **85**, 4064–4081.e4069 10.1016/j.molcel.2025.09.020 (2025). PMC12702617 41086805

[62] Park, C. et al. Antagonistic insulin derivative suppresses insulin-induced hypoglycemia. *J. Med Chem.* **66**, 7516–7522 10.1021/acs.jmedchem.3c00280 (2023). 37227951

[63] Vogel, A. et al. Structural basis of insulin receptor antagonism by bivalent site 1-site 2 ligands S961 and Ins-AC-S2. Nat. Commun. 17, 5068 10.1038/s41467-026-73851-1 (2026). PMC13249828 42265100

[64] Cao, L. et al. Design of protein-binding proteins from the target structure alone. *Nature* **605**, 551–560 10.1038/s41586-022-04654-9 (2022). PMC9117152 35332283

[65] White, M. F. A new horizon unfolding for insulin signaling in health and disease. *Mol. Cell* **85**, 4111–4113 10.1016/j.molcel.2025.10.032 (2025). 41270721

[66] Yunn, N. O. et al. A hotspot for enhancing insulin receptor activation revealed by a conformation-specific allosteric aptamer. *Nucleic Acids Res.* **49**, 700–712 10.1093/nar/gkaa1247 (2021). PMC7826266 33410883

[67] Kim, J. et al. Functional selectivity of insulin receptor revealed by aptamer-trapped receptor structures. *Nat. Commun.* **13**, 6500 10.1038/s41467-022-34292-8 (2022). PMC9618554 36310231

[68] Ashraf, A., Palakkott, A. & Ayoub, M. A. Anti-insulin receptor antibodies in the pathology and therapy of diabetes mellitus. *Curr. Diab. Rev.* **17**, 198–206 10.2174/1573399816666200604122345 (2021). 32496987

[69] Soos, M. A. et al. Monoclonal antibodies reacting with multiple epitopes on the human insulin receptor. *Biochem J.* **235**, 199–208 10.1042/bj2350199 (1986). PMC1146668 2427071

[70] Issafras, H. et al. Selective allosteric antibodies to the insulin receptor for the treatment of hyperglycemic and hypoglycemic disorders. *J. Diab. Sci. Technol.* **8**, 865–873 10.1177/1932296814529886 (2014). PMC4764207 24876415

[71] Brierley, G. V., Siddle, K. & Semple, R. K. Evaluation of anti-insulin receptor antibodies as potential novel therapies for human insulin receptoropathy using cell culture models. *Diabetologia* **61**, 1662–1675 10.1007/s00125-018-4606-2 (2018). PMC6445487 29700562

[72] Brierley, G. V. et al. Anti-insulin receptor antibodies improve hyperglycemia in a mouse model of human insulin receptoropathy. *Diabetes* **69**, 2481–2489 10.2337/db20-0345 (2020). PMC7576564 32816962

[73] Bhaskar, V. et al. A fully human, allosteric monoclonal antibody that activates the insulin receptor and improves glycemic control. *Diabetes* **61**, 1263–1271 10.2337/db11-1578 (2012). PMC3331746 22403294

[74] Bedinger, D. H., Kieffer, D. A., Goldfine, I. D., Roell, M. K. & Adams, S. H. Acute treatment with XMetA activates hepatic insulin receptors and lowers blood glucose in normal mice. *J. Cell Biochem* **116**, 2109–2119 10.1002/jcb.25168 (2015). 25808283

[75] Bezwada, P. et al. A novel allosteric insulin receptor-activating antibody reduces hyperglycemia without hypoglycemia in diabetic cynomolgus monkeys. *J. Pharm. Exp. Ther.* **356**, 466–473 10.1124/jpet.115.229690 (2016). 26578267

[76] Corbin, J. A. et al. Improved glucose metabolism in vitro and in vivo by an allosteric monoclonal antibody that increases insulin receptor binding affinity. *PLoS One* **9**, e88684 10.1371/journal.pone.0088684 (2014). PMC3922975 24533136

[77] Corbin, J. A. et al. Inhibition of insulin receptor function by a human, allosteric monoclonal antibody: a potential new approach for the treatment of hyperinsulinemic hypoglycemia. *MAbs* **6**, 262–272 10.4161/mabs.26871 (2014). PMC3929448 24423625

[78] Vajo, Z. & Duckworth, W. C. Genetically engineered insulin analogs: diabetes in the new millenium. *Pharm. Rev.* **52**, 1–9 (2000). 10699152

[79] Zaykov, A. N., Mayer, J. P. & DiMarchi, R. D. Pursuit of a perfect insulin. *Nat. Rev. Drug Discov.* **15**, 425–439 10.1038/nrd.2015.36 (2016). 26988411

[80] Sims, E. K., Carr, A. L. J., Oram, R. A., DiMeglio, L. A. & Evans-Molina, C. 100 years of insulin: celebrating the past, present and future of diabetes therapy. *Nat. Med* **27**, 1154–1164 10.1038/s41591-021-01418-2 (2021). PMC8802620 34267380

[81] Home, P. D. & Mehta, R. Insulin therapy development beyond 100 years. *Lancet Diab. Endocrinol.* **9**, 695–707 10.1016/S2213-8587(21)00182-0 (2021). 34480874

[82] Madison, L. L., Uger, R. H. & Rencz, K. The physiologic significance of secretion of insulin into portal circulation: II. Effect of rate of administration of glucagon-free insulin on magnitude of peripheral and hepatic actions. *Metabolism* **9**, 97–108 (1960). 14419686

[83] Edgerton, D. S., Moore, M. C., Gregory, J. M., Kraft, G. & Cherrington, A. D. Importance of the route of insulin delivery to its control of glucose metabolism. *Am. J. Physiol. Endocrinol. Metab.* **320**, E891–E897 10.1152/ajpendo.00628.2020 (2021). PMC8238128 33813879

[84] Li, M. et al. Trends in insulin resistance: insights into mechanisms and therapeutic strategy. *Signal Transduct. Target Ther.* **7**, 216 10.1038/s41392-022-01073-0 (2022). PMC9259665 35794109

[85] Mesika, A., Klar, A. & Falik Zaccai, T. C. in GeneReviews(*(R*)) (eds M. P. Adam *et al*.) (1993).

[86] Longo, N. et al. Impaired growth in Rabson-Mendenhall syndrome: lack of effect of growth hormone and insulin-like growth factor-I. *J. Clin. Endocrinol. Metab.* **79**, 799–805 10.1210/jcem.79.3.8077364 (1994). 8077364

[87] Quin, J. D. et al. Response of insulin-like growth factor (IGF)-binding protein-1 (IGFBP-1) and IGFBP-3 to IGF-I treatment in severe insulin resistance. *J. Endocrinol.* **141**, 177–182 10.1677/joe.0.1410177 (1994). 7516962

[88] Quin, J. D. et al. Acute response to recombinant insulin-like growth factor I in a patient with Mendenhall’s syndrome. *N. Engl. J. Med.* **323**, 1425–1426 10.1056/NEJM199011153232016 (1990). 2233914

[89] Semple, R. K., Williams, R. M. & Dunger, D. B. What is the best management strategy for patients with severe insulin resistance? *Clin. Endocrinol. (Oxf.)* **73**, 286–290 10.1111/j.1365-2265.2010.03810.x (2010). 20455892

[90] Pisani, P. Hyper-insulinaemia and cancer, meta-analyses of epidemiological studies. *Arch. Physiol. Biochem* **114**, 63–70 10.1080/13813450801954451 (2008). 18465360

[91] Pollak, M. Insulin and insulin-like growth factor signalling in neoplasia. *Nat. Rev. Cancer* **8**, 915–928 10.1038/nrc2536 (2008). 19029956

[92] Pan, F. & Hong, L. Q. Insulin promotes proliferation and migration of breast cancer cells through the extracellular regulated kinase pathway. *Asian Pac. J. Cancer Prev.* **15**, 6349–6352 10.7314/apjcp.2014.15.15.6349 (2014). 25124623

[93] Zhang, Y. et al. Oleic acid and insulin as key characteristics of T2D promote colorectal cancer deterioration in xenograft mice revealed by functional metabolomics. *Front Oncol.* **11**, 685059 10.3389/fonc.2021.685059 (2021). PMC8381473 34434893

[94] Wang, Y. et al. Uncoupling hepatic oxidative phosphorylation reduces tumor growth in two murine models of colon cancer. *Cell Rep.* **24**, 47–55 10.1016/j.celrep.2018.06.008 (2018). PMC6056247 29972790

[95] Wang, Y. et al. Insulin promotes proliferation, survival, and invasion in endometrial carcinoma by activating the MEK/ERK pathway. *Cancer Lett.* **322**, 223–231 10.1016/j.canlet.2012.03.026 (2012). 22459351

[96] Gallagher, E. J. & LeRoith, D. The proliferating role of insulin and insulin-like growth factors in cancer. *Trends Endocrinol. Metab.* **21**, 610–618 10.1016/j.tem.2010.06.007 (2010). PMC2949481 20663687

[97] Vigneri, R., Goldfine, I. D. & Frittitta, L. Insulin, insulin receptors, and cancer. *J. Endocrinol. Invest* **39**, 1365–1376 10.1007/s40618-016-0508-7 (2016). 27368923

[98] Milazzo, G., Sciacca, L., Papa, V., Goldfine, I. D. & Vigneri, R. ASPB10 insulin induction of increased mitogenic responses and phenotypic changes in human breast epithelial cells: evidence for enhanced interactions with the insulin-like growth factor-I receptor. *Mol. Carcinog.* **18**, 19–25 10.1002/(sici)1098-2744(199701)18:1<19::aid-mc3>3.0.co;2-m (1997). 9022809

[99] Hvid, H. et al. Stimulation of MC38 tumor growth by insulin analog X10 involves the serine synthesis pathway. *Endocr. Relat. Cancer* **19**, 557–574 10.1530/ERC-12-0125 (2012). 22685267

[100] Hvid, H. et al. Treatment with insulin analog X10 and IGF-1 increases growth of colon cancer allografts. *PLoS One* **8**, e79710 10.1371/journal.pone.0079710 (2013). PMC3832545 24260289

[101] An, W. et al. Activation of the insulin receptor by insulin-like growth factor 2. *Nat. Commun.* **15**, 2609 10.1038/s41467-024-46990-6 (2024). PMC10960814 38521788

[102] Belfiore, A. & Malaguarnera, R. Insulin receptor and cancer. *Endocr. Relat. Cancer* **18**, R125–R147 10.1530/ERC-11-0074 (2011). 21606157

[103] Frasca, F. et al. Insulin receptor isoform A, a newly recognized, high-affinity insulin-like growth factor II receptor in fetal and cancer cells. *Mol. Cell Biol.* **19**, 3278–3288 10.1128/MCB.19.5.3278 (1999). PMC84122 10207053

[104] Vella, V. et al. A novel autocrine loop involving IGF-II and the insulin receptor isoform-A stimulates growth of thyroid cancer. *J. Clin. Endocrinol. Metab.* **87**, 245–254 10.1210/jcem.87.1.8142 (2002). 11788654

[105] Cassar, S. et al. Insulin resistance in polycystic ovary syndrome: a systematic review and meta-analysis of euglycaemic-hyperinsulinaemic clamp studies. *Hum. Reprod.* **31**, 2619–2631 10.1093/humrep/dew243 (2016). 27907900

[106] Rojas, J. et al. Polycystic ovary syndrome, insulin resistance, and obesity: navigating the pathophysiologic labyrinth. *Int J. Reprod. Med.* **2014**, 719050 10.1155/2014/719050 (2014). PMC4334071 25763405

[107] Dunaif, A. Insulin resistance and the polycystic ovary syndrome: mechanism and implications for pathogenesis. *Endocr. Rev.* **18**, 774–800 10.1210/edrv.18.6.0318 (1997). 9408743

[108] Dunaif, A. et al. Evidence for distinctive and intrinsic defects in insulin action in polycystic ovary syndrome. *Diabetes* **41**, 1257–1266 10.2337/diab.41.10.1257 (1992). 1397698

[109] Book, C. B. & Dunaif, A. Selective insulin resistance in the polycystic ovary syndrome. *J. Clin. Endocrinol. Metab.* **84**, 3110–3116 10.1210/jcem.84.9.6010 (1999). 10487672

[110] Corbould, A. et al. Insulin resistance in the skeletal muscle of women with PCOS involves intrinsic and acquired defects in insulin signaling. *Am. J. Physiol. Endocrinol. Metab.* **288**, E1047–E1054 10.1152/ajpendo.00361.2004 (2005). 15613682

[111] Dunaif, A., Wu, X., Lee, A. & Diamanti-Kandarakis, E. Defects in insulin receptor signaling in vivo in the polycystic ovary syndrome (PCOS). *Am. J. Physiol. Endocrinol. Metab.* **281**, E392–E399 10.1152/ajpendo.2001.281.2.E392 (2001). 11440917

[112] Wu, X. et al. Expression of insulin-receptor substrate-1 and -2 in ovaries from women with insulin resistance and from controls. *Fertil. Steril.* **74**, 564–572 10.1016/s0015-0282(00)00688-9 (2000). 10973656

[113] Wu, X. K. et al. Selective ovary resistance to insulin signaling in women with polycystic ovary syndrome. *Fertil. Steril.* **80**, 954–965 10.1016/s0015-0282(03)01007-0 (2003). 14556818

[114] Yen, H. W., Jakimiuk, A. J., Munir, I. & Magoffin, D. A. Selective alterations in insulin receptor substrates-1, -2 and -4 in theca but not granulosa cells from polycystic ovaries. *Mol. Hum. Reprod.* **10**, 473–479 10.1093/molehr/gah066 (2004). 15155816

[115] Han, J. M., Patterson, S. J., Speck, M., Ehses, J. A. & Levings, M. K. Insulin inhibits IL-10-mediated regulatory T cell function: implications for obesity. *J. Immunol.* **192**, 623–629 10.4049/jimmunol.1302181 (2014). 24323581

[116] Mauer, J. et al. Myeloid cell-restricted insulin receptor deficiency protects against obesity-induced inflammation and systemic insulin resistance. *PLoS Genet* **6**, e1000938 10.1371/journal.pgen.1000938 (2010). PMC2865520 20463885

[117] Tsai, S. et al. Insulin receptor-mediated stimulation boosts T cell immunity during inflammation and infection. *Cell Metab.* **28**, 922–934.e924 10.1016/j.cmet.2018.08.003 (2018). 30174303

[118] Belfiore, A. et al. Insulin receptor isoforms in physiology and disease: an updated view. *Endocr. Rev.* **38**, 379–431 10.1210/er.2017-00073 (2017). PMC5629070 28973479

[119] Malaguarnera, R. et al. Comparative proteomic analysis of insulin receptor isoform A and B signaling. *Mol. Cell Endocrinol.* **557**, 111739 10.1016/j.mce.2022.111739 (2022). 35940390

[120] de la Monte, S. M. & Tong, M. Brain metabolic dysfunction at the core of Alzheimer’s disease. *Biochem Pharm.* **88**, 548–559 10.1016/j.bcp.2013.12.012 (2014). PMC4550323 24380887

[121] Kellar, D. & Craft, S. Brain insulin resistance in Alzheimer’s disease and related disorders: mechanisms and therapeutic approaches. *Lancet Neurol.* **19**, 758–766 10.1016/S1474-4422(20)30231-3 (2020). PMC9661919 32730766

[122] Chiu, S. L., Chen, C. M. & Cline, H. T. Insulin receptor signaling regulates synapse number, dendritic plasticity, and circuit function in vivo. *Neuron* **58**, 708–719 10.1016/j.neuron.2008.04.014 (2008). PMC3057650 18549783

[123] Talbot, K. et al. Demonstrated brain insulin resistance in Alzheimer’s disease patients is associated with IGF-1 resistance, IRS-1 dysregulation, and cognitive decline. *J. Clin. Invest* **122**, 1316–1338 10.1172/JCI59903 (2012). PMC3314463 22476197

[124] Bomfim, T. R. et al. An anti-diabetes agent protects the mouse brain from defective insulin signaling caused by Alzheimer’s disease- associated Abeta oligomers. *J. Clin. Invest* **122**, 1339–1353 10.1172/JCI57256 (2012). PMC3314445 22476196

[125] Zhao, N. et al. Apolipoprotein E4 impairs neuronal insulin signaling by trapping insulin receptor in the endosomes. *Neuron* **96**, 115–129.e115 10.1016/j.neuron.2017.09.003 (2017). PMC5621659 28957663

[126] Gralle, M., Labrecque, S., Salesse, C. & De Koninck, P. Spatial dynamics of the insulin receptor in living neurons. *J. Neurochem* **156**, 88–105 10.1111/jnc.14950 (2021). 31886886

[127] Yu, M. et al. Battle of GLP-1 delivery technologies. *Adv. Drug Deliv. Rev.* **130**, 113–130 10.1016/j.addr.2018.07.009 (2018). PMC6843995 30009885

